# Correlation Between Peak Expiratory Flow Rate, Markers of Adiposity, and Anthropometric Measures in Medical Students in Pakistan

**DOI:** 10.7759/cureus.12408

**Published:** 2020-12-31

**Authors:** Amna Ijaz, Imtiaz Bashir, Azal Ikhlaq, Farhat Ijaz, Rana Khurram Aftab, Robass Zia

**Affiliations:** 1 Psychiatry, Nawaz Shareef Medical College, Lahore, PAK; 2 Medicine, Combined Military Hospital (CMH) Lahore Medical College and Institute of Dentistry, Lahore, PAK; 3 Physiology, Combined Military Hospital (CMH) Lahore Medical College and Institute of Dentistry, Lahore, PAK; 4 Physiology, King Edward Medical University, Lahore, PAK; 5 Physiology, Combined Military Hospital (CMH) Medical College, Lahore, PAK

**Keywords:** obesity and lung function, pefr and bmi, pefr and whr, pefr and gender, pefr and anthropometric measures

## Abstract

Obesity has been defined as the excessive deposition of fats on the body. It presents a very significant risk to humanity, with debilitating consequences for healthcare systems worldwide. It has multiple effects on the body, including grave consequences on the cardiovascular and respiratory systems. Our project explores the latter. There are multiple studies available in the scientific literature that attempt to explain this phenomenon, all with limited success and conflicting results.

This cross-sectional exploration of the topic was done on medical undergraduates to pick up on any correlations between peak expiratory flow rate (PEFR) and the markers of obesity (body mass index (BMI), waist circumference (WC), hip circumference (HC), and waist to hip ratio (WHR)).

In general, we found that male participants had sizably higher PEFR values than females (r=0.540, p<0.01). Appropriate BMI is mandatory for the physiologic functioning of the human body. This work also statistically demonstrates a negative overall correlation between lung health and various parameters of obesity. Our work suggests a positive correlation between WHR and PEFR (r=0.325, p<0.01), BMI and PEFR (r=0.573, p=0.02), along with weight and PEFR (r=0.464, p<0.01). Maintaining a BMI and WHR in the normal range is essential for optimal physiological functioning and physical well-being.

## Introduction

“Abnormal or excessive accumulation of fats in the body that might be harmful to an individual is termed as obesity [1].” Work done by the World Health Organization shows that global obesity rates have more or less tripled since 1975. As of 2016, more than a third of adults are overweight while nearly a tenth are obese [2]. Obesity is a significant health risk; its prevalence now indicates a near-pandemic. Obesity dramatically heightens the risk of diseases like type II diabetes, myocardial infarction, and various types of cancers. It is thus hazardous to both the quality and expectancy of life [3]. A multitude of factors such as food habits, lifestyle, and the complicated interactions between genetic, cultural, and socioeconomic forces are all fundamental causes of obesity [4]. Several studies conclude that obesity is also rampant in medical students [5-6]. Recent scholarly work done in Karachi, Pakistan, similarly demonstrates that nearly a third of medical students can be classified as obese [7].

Body mass index (BMI), hip circumference (HC), waist circumference (WC), and waist to hip ratio (WHR) are the markers of obesity and are useful for judging the outcomes of cardiovascular co-morbidities [8].

Correlations between obesity and pulmonary function have been studied in the past, however, no universal consensus on the mechanisms that lead to respiratory complications has been reached. One leading hypothesis posits that increased adiposity in the thoracoabdominal region limits the movement of the musculature of the region and thus may impair appropriate lung mobility and hence decrease ventilation. Furthermore, adipose tissue, due to its paracrine and endocrine functions, secretes cytokines and other bioactive mediators that are pro-inflammatory. These substances have been associated with lung hypo-development, decreased bronchial responsiveness, and, eventually, atrophy [9].

Pulmonary function tests (PFT) are a basic tool for assessing lung dysfunction, disease, and prognosis of treatment. Peak expiratory flow rate (PEFR) is the maximum speed of airflow attained by a forceful, complete expiration after complete inhalation. It is usually measured using a portable instrument called Wright’s peak flow meter. While forced expiratory volume after the first second of exhalation (FEV1) is the gold-standard test for pulmonary function, it is not always a plausible option. Additionally, the peak flow meter is a reliable and easy-to-use tool [10].

Prior studies have suggested conflicting links for the relationship between obesity and PEFR [11-14]. Our research aims to assess the correlation between the obesity markers (i.e. BMI, WC, HC, WHR) and PEFR. The results will be helpful in the care of overweight patients with respiratory and cardiovascular co-morbidities.

## Materials and methods

This cross-sectional study was done on first-year medical undergraduates of CMH Lahore Medical College, Lahore, Pakistan, in November 2019. Ethical approval was taken from the Ethical Review Committee of the same institution prior to the conduction of this study. One-hundred thirty-eight (138) students of first-year MBBS (73 males and 65 females) took part in the research. Students with a current history of disease, infection, or allergy were excluded. Informed, written consent was collected from all the participants before the experiment was conducted. Demographic details were taken, and the procedure was explained clearly. The data were recorded in the observation sheet of the research team. Participants were weighed using calibrated Libra weighing scales and their heights were recorded in centimeters using height measuring rods. Individual BMI was determined using:

 BMI = Weight (kg)/Height (m^2^)

Participants were classified on the basis of their BMI values. Underweight (BMI<18.5), normal weight (18.5≤BMI≤24.9), overweight (25≤BM≤29.9), and obese (BMI≥30). Waist circumference was taken using a non-flexible, measurement tape held horizontally after a normal expiration in the anatomical position. Hip circumference was calculated in centimeters at the point of the most significant width of the hip. All the participants were taught the procedure for appropriate use of the peak flow meter and were asked to practice using it before the actual experiment. PEFR readings were repeated thrice using Wright’s peak flow meter. The highest of the three values was considered.

The relevant data were recorded and studied using the Statistical Package for Social Sciences version 25.0 (IBM Corp., Armonk, NY). Pearson’s correlation was used to determine the correlation between the variables. A p-value less than 0.05 using a two-tailed significance test was held to be statistically significant.

## Results

The anthropometric data and PEFRs of participants categorized based on gender are shown in Table 1.

**Table 1:** Anthropometric data and PEFRs of participants categorized based on gender BMI=Body Mass Index, PEFR=Peak Expiratory Flow Rate

Parameter Mean + S.D	Male (n = 73)	Female (n = 65)
Age (Years)	18.93+1.04	18.88+0.99
Height (cm)	173.28+6.67	158.62+7.93
Weight (kg)	68.13+10.73	58.08+14.49
BMI (kg/m^2^)	22.65+3.11	23.16+6.01
Waist Circumference (cm)	80.22+9.92	74.32+11.69
Hip Circumference (cm)	95.03+10.35	95.06+11.27
Waist/Hip Ratio	0.844+0.04	0.781+0.05
PEFR (L/second)	433.97+101.84	325.23+62.30

Table 2 presents correlations between two of the tested variables, with their p-values. It is evident that a statistically important positive correlation exists between PEFR and WHR (r=0.325, p=0.00), PEFR and height (r=0.464, p=0.00), and PEFR and gender (r=0.540, p=0.00). Overall, males presented with higher PEFR readings than females.

**Table 2:** Pearson correlations between PEFR and other variables of all the participants Note: * donates a statistically significant correlation between the two given variables

Parameters	Correlation	P-Value
Waist Circumference and Peak Expiratory Flow Rate	0.14	0.103
Hip Circumference and Peak Expiratory Flow Rate	-0.025	0.771
Waist to Hip Ratio and Peak Expiratory Flow Rate	0.325*	0.000
Body Mass Index and Peak Expiratory Flow Rate	0.051	0.551
Gender and Peak Expiratory Flow Rate	-0.540*	0.000
Height and Peak Expiratory Flow Rate	0.464*	0.000

Table 3 presents the degree of correlations categorically for males. 

**Table 3:** Pearson correlations between PEFR and other variables of the male participants PEFR=Peak Expiratory Flow Rate

Parameters	Correlation	P-Value
Waist Circumference and Peak Expiratory Flow Rate	-0.016	0.893
Hip Circumference and Peak Expiratory Flow Rate	-0.025	0.831
Waist to Hip Ratio and Peak Expiratory Flow Rate	0.061	0.608
Body Mass Index and Peak Expiratory Flow Rate	0.191	0.105
Height and Peak Expiratory Flow Rate	0.180	0.127

Table 4 presents the degree of correlations categorically for females. There is no quantitatively significant correlation between the variables when taken categorically for the genders.

**Table 4:** Pearson correlations between PEFR and other variables of the female participants PEFR=Peak Expiratory Flow Rate

Parameters	Correlation	P-Value
Waist Circumference and Peak Expiratory Flow Rate	0.013	0.919
Hip Circumference and Peak Expiratory Flow Rate	-0.037	0.769
Waist to Hip Ratio and Peak Expiratory Flow Rate	0.073	0.562
Body Mass Index and Peak Expiratory Flow Rate	0.038	0.764
Height and Peak Expiratory Flow Rate	0.082	0.516

Table 5 gives the weight status of the participants. Only three out of 138 were obese while 15 were underweight and 35 were overweight. A statistically significant correlation was found between PEFT and BMI in the underweight and normal-weight grouped people (r=0.573, p=0.02 in underweight people, and r=0.240, p=0.02 in normal-weight people). However, no significant correlation between BMI and PEFR was found in overweight and obese people.

**Table 5:** Weight status of the participants based upon BMI BMI=Body Mass Index

Weight Status (BMI)	Cumulative n(%)	Male n (%)	Female n (%)
Underweight (<18.5)	15 (10.9%)	5 (6.8%)	10 (15.4%)
Normal Weight (18.5-24.9)	85 (61.6%)	50 (68.5%)	35 (53.8%)
Overweight (25-29.9)	35 (25.4%)	18 (24.7%)	17 (26.2%)
Obese (>30)	3 (2.2%)	-	3 (4.6%)
	n=138	n=73	n=65

## Discussion

Obesity is an emerging problem in Pakistan, not least in women and young adults. This is owing to sedentary lifestyles and unhealthy food habits [15]. Our study demonstrates a significant link between obesity markers and lung function tests (PEFR) in medical undergraduates. As a whole, PEFR in males was greater than in females (p<0.01). One explanation for this finding is based on height. A statistically significant relationship (p<0.01) is also found between height and PEFR when studied for the combined data of both genders. In our study population, males were generally taller than females (the mean height of males was 173.28+6.67 cm and of the females was 158.62+7.93 cm). Given that (1) height is positively related to PEFR and (2) males are taller in this study population, this might account for the higher PEFR findings in males than females. Similar findings have been reported previously [16]. An alternate explanation suggests that decreased PEFR in females might also be associated with decreased physical activity and ethnic background, but this is limited in its explanatory powers and was not tested in our work and thus cannot be objectively remarked upon without further study.

A positive correlation was found between the waist to hip ratio (WHR) and PEFR (p<0.01) when the combined data were studied, however, no correlation was found in the data categorized for genders. This finding is in contrast with a previous study [17], which suggested that WHR and WC affect the PEFR in males but not in females, and other anthropometric measures such as height do not affect it. We believe our work supersedes this study, as we have conducted a more thorough statistical analysis. Some studies concur with their conclusions [11,13]. These studies hypothesize that the body fat arrangement might adversely affect PEFR because of adiposity patterning differences in males and females; because there is greater central obesity in males, there is thus a more significant external pressure on the diaphragm, which leads to decreased lung functionality.

However, our data suggest the opposite since no correlation was found between PEFR and WC and between PEFR and HC in either the combined or categorical data. However, the W/H ratio is positively associated with PEFR in the combined data but not in the categorical data. A balanced W/H ratio might be positively associated with PEFR because when the data were categorized by the weight status of the participants, this correlation was significant (r=0.453, p=0.00) only in the normal-weight people. However, we would strongly suggest follow-up studies to confirm and corroborate these findings.

Another statistically significant positive correlation (p<0.05) was between PEFR and BMI. This finding is consistent with a previous study done on medical students in Karachi, Pakistan [18]. However, this association was found only in underweight and normal-weight people. There is a positive correlation between PEFR and BMI but only in underweight and normal-weight people. No statistically sound correlation was found in the other categories. Thus, we can conclude that increasing the BMI within normal limits might improve PEFR and thus lung function and quality of life. This gives further proof to the already established finding that physiologically healthy BMI levels are necessary for the healthy functioning of humans [19].

Despite the astute and practical discoveries of this project, there were limitations to our methodology and equipment. One limitation is the use of the peak flow meter to assess PEFR values. More sophisticated tools would have allowed for more accurate readings and more generalizable findings. Increasing sample size would have benefited us to the same effect as prior. We recommend a larger-scale survey to further validate our work.

## Conclusions

A positive correlation exists between WHR and PEFR but only in people with normal BMI. PEFR is also correlated positively with BMI but only in underweight and normal-weight people. There is also a significant positive correlation between height and PEFR. Additionally, male participants have a higher PEFR value than females. Maintaining a BMI and WHR in the normal range is essential for optimal physiological functioning and physical well-being.
